# Bioprospecting of Less-Polar Constituents from Endemic Brown Macroalga *Fucus virsoides* J. Agardh from the Adriatic Sea and Targeted Antioxidant Effects In Vitro and In Vivo (Zebrafish Model)

**DOI:** 10.3390/md19050235

**Published:** 2021-04-22

**Authors:** Igor Jerković, Ana-Marija Cikoš, Sanja Babić, Lara Čižmek, Krunoslav Bojanić, Krunoslav Aladić, Nikolay V. Ul’yanovskii, Dmitry S. Kosyakov, Albert T. Lebedev, Rozelindra Čož-Rakovac, Polonca Trebše, Stela Jokić

**Affiliations:** 1 Department of Organic Chemistry, Faculty of Chemistry and Technology, University of Split, Ruđera Boškovića 35, 21000 Split, Croatia; 2 Department of Process Engineering, Faculty of Food Technology, Josip Juraj Strossmayer University of Osijek, Franje Kuhača 18, 31000 Osijek, Croatia; acikos@ptfos.hr (A.-M.C.); kaladic@ptfos.hr (K.A.); 3 Laboratory for Aquaculture Biotechnology, Division of Materials Chemistry, Ruđer Bošković Institute, Bijenička cesta 54, 10000 Zagreb, Croatia; sanja.babic@irb.hr (S.B.); lara.cizmek@irb.hr (L.Č.); krunoslav.bojanic@irb.hr (K.B.); rozelindra.coz-rakovac@irb.hr (R.Č.-R.); 4 Laboratory of Environmental Analytical Chemistry, Core Facility Center “Arktika”, Northern (Arctic) Federal University, Naberezhnaya Severnoy Dviny 17, 163002 Arkhangelsk, Russia; n.ulyanovsky@narfu.ru (N.V.U.); d.kosyakov@narfu.ru (D.S.K.); 5 Department of Organic Chemistry, Lomonosov Moscow State University, 119991 Moscow, Russia; mocehops@ya.ru; 6 Faculty of Health Sciences, University of Ljubljana, Zdravstvena pot 5, 1000 Ljubljana, Slovenia; polonca.trebse@zf.uni-lj.si

**Keywords:** brown algae, volatiles, fatty acids, pigments, bioassays, embryotoxicity, reactive oxygen species

## Abstract

The endemic brown macroalga *Fucus virsoides* J. Agardh from the Adriatic Sea was in the focus of the present research. The volatiles of fresh (FrFv) and air-dried (DrFv) samples of *F. virsoides* obtained by headspace solid-phase microextraction (HS-SPME) and hydrodistillation (HD) were analyzed by gas chromatography equipped with flame ionization detector and mass spectrometry (GC-FID/MS). The major HS-FrFv compound was pentadecane (61.90–71.55%) followed by pentadec-1-ene (11.00–7.98%). In HS-DrFv, pentadec-1-ene was not present, and few lower aliphatic compounds appeared, as well as benzaldehyde and benzyl alcohol. In HD-FrFv, particularly abundant were alkenes (such as pentadec-1-ene (19.32%), or (*E*)-pentadec-7-ene (8.35%)). In HD-DrFv, more oxidation products were present (e.g., carbonyl compounds such as tridecanal (18.51%)). The fatty acids profile of freeze-dried sample (FdFv) after conversion to methyl esters was determined by GC-FID, and oleic acid was dominant (42.28%), followed by arachidonic acid (15.00%). High-performance liquid chromatography-high-resolution mass spectrometry with electrospray ionization (HPLC-ESI-HRMS) was used for the screening of less polar fractions (F3 and F4) of *F. virsoides*. Mono- and diglycerides of stearic, palmitic, oleic, and arachidonic acids were found. Terpenoids and steroids comprised the compounds C_20_H_30(32)_O_2_ and C_29_H_48_O_(2)_. Among carotenoids, fucoxanthin was identified. Chlorophyll derivatives were also found (C_55_H_74(72)_N_4_O_(5-7)_), dominated by pheophytin *a*. The antioxidant activity of the fractions was investigated by in vitro assays (oxygen radical absorbance capacity (ORAC), reduction of radical cation (ABTS•^+^), 2,2-diphenyl-1-picryl-hydrazyl-hydrate (DPPH) assay, and ferric reducing antioxidant power (FRAP)) and by in vivo zebrafish model (along with fish embryotoxicity). In vitro experiments proved good radical scavenging abilities of F3 and F4 fractions, which were additionally supported by the protective effect against hydrogen peroxide-induced oxidative stress in zebrafish embryos.

## 1. Introduction

Macroalgae of the genus *Fucus* have been a valuable source of bioactive components, containing complex polysaccharides, polyphenols, fatty acids, and vitamins [1]. The focus of our continuous investigation on the Bioprospecting of the Adriatic Sea in Croatia in the present research is on *Fucus virsoides* J. Agardh from the family Fucaceae, order Fucales as an endemic brown macroalga distributed only in the mediolittoral zones on rocky sheltered or moderately exposed shores only in the Adriatic Sea [2].

*F. virsoides* was previously investigated with respect to basic physiological features (dark carbon fixation, photosynthesis, and respiration) [2,3]. There are few papers on the phytochemical composition of *F. virsoides* that evidenced the presence of triacylglycerols, fucosterol, galactosyldiacylglycerols, and fucoxanthin [4]. Sulphated polysaccharides were also isolated, and after total acid hydrolysis, fucose was the dominant carbohydrate in *F. virsoides*, followed by xylose, mannose, and galactose [5]. In the sterol fraction, only fucosterol, which made up to 92% of the total sterols, was found [6], which is characteristic for brown algae, and particularly for the genus *Fucus* (e.g., *F. evanescens, F. serratus*, and *F. vesiculosus*). The effect of the substratum type on the fatty acid composition of *F. virsoides* in six habitats was investigated [7]. *F. virsoides* fall fraction contained a better ratio of polyunsaturated fatty acids (PUFAs) to saturated fatty acids (SFAs) as well as higher ω6 and ω3 fatty acids content in comparison with the summer fraction [8]. In both fractions, the most abundant SFA was palmitic acid, followed by myristic acid, stearic acid, and pentadecanoic acid; lauric acid was only found in the fall fraction. The dominant monounsaturated fatty acid (MUFA) in both fractions was oleic acid, while palmitoleic acid content was remarkably lower. Arachidonic acid, α-linolenic acid, and linoleic acid were the most abundant PUFAs in both fractions, while dihomo-γ-linolenic acid and eicosadienoic acid were detected only in the fall fraction. The two fall fractions (ethyl acetate and petroleum ether) loaded with polyphenols, and PUFAs exhibited anticancer activity (proapoptotic activity for human cervical adenocarcinoma HeLa cells) anti-α-glucosidase activity [8], and better suppression of EA.hy926 cells migration and tube formation [8]. Ethyl acetate fractions showed the best antibacterial and antifungal activity on *Staphylococcus aureus, Bacillus cereus, Bacillus subtilis, Proteus mirabilis*, and *Escherichia coli* [8].

Although the mentioned papers report the phytochemical composition for targeted compounds, we found a gap in the volatile organic compounds (VOCs) that were not investigated so far in *F. virsoides*. Since the volatile hydrocarbons (e.g., expected pentadecane as characteristic compounds of brown algae [9,10,11]) have been connected to fatty acids degradation, we reexamined their composition in the collected sample. This was necessary since it has been reported that their composition has been influenced by the collection period, geographic location, and salinity [8]. The determination of fatty acids in brown algae is crucial not only to investigate and compare metabolic pathways, but also to assess the possible source of PUFAs such as ω3 and ω6, some of which are considered essential nutrients [12] that exhibit different biological activities [13]. In addition, the targets of our research are non-volatile less polar compounds and their possible in vitro and in vivo antioxidant capacity (first comprehensive study) since they could contain bioactive compounds that scavenge free radicals, thus preventing oxidation-linked diseases i.e., cancer, heart disease, atherosclerosis, aging processes, etc. [14].

Therefore, the main goals of the present study on *F. virsoides* were to: (a) explore and compare VOCs of fresh (FrFv) and air-dried (DrFv) *F. virsoides* isolated by hydrodistillation (HD) and headspace solid-phase microextraction (HS-SPME) followed by gas chromatography analysis with flame ionization detector and mass spectrometry (GC-FID/MS); (b) identify fatty acids present in freeze-dried sample (FdFv) by GC-FID analysis after their conversion to methyl esters; (c) screen non-targeted less polar non-volatiles of semi-purified fractions of FdFv by high-performance liquid chromatography-high-resolution mass spectrometry with electrospray ionization (HPLC-ESI-HRMS); (d) comprehensively evaluate the antioxidant activity of the fractions by four in vitro assays (2,2-diphenyl-1-picryl-hydrazyl-hydrate (DPPH) assay, reduction of radical cation ABTS•^+^, oxygen radical absorbance capacity (ORAC), and ferric reducing antioxidant power (FRAP)) and in vivo zebrafish model (along with zebrafish embryotoxicity); (e) test the antimicrobial activity of semi-purified less-polar fractions of FdFv.

## 2. Results and Discussion

### 2.1. Volatile Organic Compounds (VOCs) Analysis

To obtain more and less volatile organic compounds as well as the headspace compounds (first-time report) of *F. virsodes*, two methods were used: HS-SPME and HD combined with GC-FID/MS analysis. PDMS/DVB and DVB/CAR/PDMS fibers were used for exploring detailed headspace composition. Significant differences were found among the HS-SPME and HD profiles of the fresh sample, as well as among the profiles of fresh and dried *F. virsoides*.

#### 2.1.1. The Headspace Composition

The major compound of the headspace of fresh *F. virsoides* (HS-FrFv) was pentadecane (61.90–71.55%), followed by pentadec-1-ene (11.00–17.98%). It is already known [9,10,11] that *n*-pentadecane predominates in the brown algae and *n*-heptadecane in the red algae. However, the brown algae hydrocarbons vary remarkably (from almost saturated to fully olefinic), but the alkane/alkene ratios must be interpreted with caution due to the large variation, even within various parts of the same alga [9]. Two major compounds, (*E*)-pentadec-7-ene (only found by DVB/CAR/PDMS fiber) and tridecanal (only identified by PDMS/DVB fiber), were found. Benthic marine algae unsaturated and saturated hydrocarbon content (based on a dry weight) was determined [11] by the Soxhlet extraction for *F. distichus* (99% for saturated hydrocarbons (SH) and 0.1% for unsaturated hydrocarbons (USH)), *F. spiralis* (96.5% for SH and 3.5% for USH), and *F. vesiculosis* (81.6% for SH and 18.4% for USH). Pentadecane and pentadec-1-ene were found in *F. distichus* (98%; 0.1%), *F. spiralis* (95%; 0.2%), and *F. vesiculosis* (65%; 0.4%). Therefore, there is a similarity with our results on HS-FrFv and HS-DrFv (Table 1).

Drying of the alga influenced the headspace composition (HS-DrFv). Although the major compound pentadecane was in HS-DrFv within the similar range (60.27–71.43%) as in HS-FrFv, pentadec-1-ene was not present in HS-DrFv. Its volatility (boiling point 268.7 °C) is close to pentadecane (270.6 °C), with a similar core alkane backbone as pentadecane, and it can be assumed that it was oxidative degraded (e.g., to tridecane found only in HS-DrFv) during drying rather than evaporated, taking into account that pentadecane remained the predominant compound of the headspace. In addition, new compounds appeared: aromatic compounds (benzyl alcohol 19.67%; 15.75% and benzaldehyde 1.43%; 1.18%) and lower aliphatic carbonyl compounds (hexanal, heptanal, (*E*)-hept-2-enal, oct-1-en-3-ol, oct-1-en-3-one, octanal and nonanal). The origin of lower aliphatic carbonyl compounds during drying may be related to pronounced oxidation and decomposition reactions of lipid-derived compounds, as was noted in our other research on the algae [15]. On the other hand, the appearance of benzaldehyde during drying under conditions of lipid oxidation and air access can be connected with a recent model study [16]. The study showed that benzaldehyde and phenylacetic acid can occur by the chemical routes of lipid oxidation [16]. Their occurrence is complex and involves carbonyl-amine reactions (or Maillard reactions) in the first stage to produce phenylacetaldehyde, phenylpyruvic acid, or β-phenylethylamine, which were further degraded by the free radicals from the lipid hydroperoxides decomposition to form benzaldehyde and phenylacetic acid. Phenylacetaldehyde was the main origin of benzaldehyde [16], mainly at air and acidic pH and in the presence of either lipid hydroperoxides (LOOH) or the mixture of 4-oxo-non-2-enal (ON) and LOOH). A notable content of benzaldehyde was also produced by phenylpyruvic acid degradation (under similar conditions), although to a lower extent than was noticed for phenylacetaldehyde. Approximately 10–15% of overall chromatogram peaks percentage area of HS-FrFv and HS-DrFv was not identified.

#### 2.1.2. The Volatile Oil Composition

Higher aliphatic compounds were the major constituents of hydrodistillate of fresh *F. virsoides* (HD-FrFv). Particularly abundant were alkenes (pentadec-1-ene (19.32%), (*E*)-pentadec-7-ene (8.35%), (*Z*)-heptadec-3-ene (6.07%), heptadec-1-ene (5.05%), and (*E*)-heptadec-8-ene (0.61%)) and alkanes (pentadecane (5.87%) and heptadecane (0.95%)). In distinction from the headspace (Table 1), pentadecane was significantly less represented in HD-FrFv while the abundance of pentadec-1-ene was slightly elevated (Table 2). Elongation-decarboxylation pathway in the algae [17] have found decarboxylases capable of transformation of the fatty acid to alkane. Several mechanisms have been suggested, including an intermediate vinyl group formation, which may further be reduced to the corresponding alkane (like pentadec-1-ene and heptadec-1-ene) and direct decarboxylation to an alkane.

Fatty acids were minor constituents in HD-FrFv (Table 2), but they were determined in the sample after derivatization (segment 2.2). Linolenic alcohol was found in HD-FrFv at 12.89%. Tridecanal, tetradecanal, decadienal isomers, and nonanal were present as minor constituents (Table 2).

Phytol ((*E*)-3,7,11,15-tetramethylhexadec-2-enol; the ester-linked side-chain of chlorophyll *a*) was present at 7.19%. Its origin in the marine environment can be related to chlorophyll phytyl chain photodegradation, hydroperoxide-induced oxidation, or other degradations [18]. Chlorophyll *a* photodegradation in seawater directs the formation of relatively stable photoproducts, which afforded 6,10,14-trimethylpentadecan-2-one (hexahydrofarnesyl acetone) that was found in HD-FrFv at 2.23%. In the marine environment, this bounded ketone was suggested as a marker for the photodegradation of chlorophylls [19].

Among minor constituents from other classes of natural compounds, aromatic compounds (benzaldehyde, benzyl alcohol, phenylacetaldehyde, and benzothiazole) and norisoprenoids (4-ketoisophorone and β-ionone) were present.

The chemical profile of HD-DrFv was different than HD-FrFv, containing more oxidation products among identified VOCs (e.g., carbonyl compounds not found in HD-FrFv). In total, 25 compounds were exclusively present in HD-DrFv. The major compound of HD-DrFv was tridecanal (18.51%), with the percentage ca. 7 times higher than in HD-FrFv. It could have arisen by oxidation of the double bond of the corresponding fatty acids present in the sample during drying. In addition, minor elevated percentages of pentadecanal and tetradecanoic acid were found. Lower aliphatic compounds found only in HD-DrFv were aldehydes: (*E*)-hex-2-enal, heptanal, (*E*,*E*)-hepta-2,4-dienal, octanal, (*E*,*Z*)-hepta-2,4-dienal, (*Z*,*Z*)-hepta-2,4-dienal, (*E*)-oct-2-enal, (*E*,*E*)-nona-2,6-dienal, (*E*)-non-2-enal, decanal, and dodecanal. The aldehydes present in HD-FrFv ((*Z*)-hept-2-enal, nonanal and (*E*,*Z*)-deca-2,4-dienal) were found in HD-DrFv with elevated percentages. The unsaturated aldehydes probably arose from the cleavage of the double bonds of corresponding unsaturated fatty acids (Table 3) or linolenic alcohol (whose percentage was reduced in HD-DrFv, Table 2) during drying. Aliphatic ketones were also found (octane-2,3-dione, (*E*,*E*)-octa-3,5-dien-2-one, nonan-2-one, and (*E*,*Z*)-octa-3,5-dien-2-one) as well as aliphatic alcohols ((*E*)-oct-2-en-1-ol, oct-1-en-3-ol, and octan-1-ol). The abundance of benzyl alcohol, phenylacetaldehyde, and benzaldehyde was increased (Table 2) in HD-DrFv, which was discussed in Section 2.1.1. Norisoprenoids percentages were elevated in HD-DrFv for 4-ketoisophorone (ca. 1.5 times) and β-ionone (ca. 7.5 times), while α-ionone appeared (0.79%) in HD-DrFv, indicating carotenoid degradation during drying.

### 2.2. Analysis of Fatty Acids

Determined fatty acids (FA) of *F. virsoides* after derivatization and GC-FID analysis are presented in Table 3, and 21 fatty acids were found in FdFv.

The main fatty acids found (Table 3) were oleic acid isomers (C18:1n9t + C18:1n9c) as the dominant, accompanied by arachidonic acid (C20:4n6) and myristic acid (C14:0) with 42.28%, 15.00%, and 10.51%, respectively. The percentage of SFAs was 22.03%. The content of unsaturated fatty acids was more dominant: 44.43% of MUFAs followed by 33.71% of PUFAs. PUFAs are known for their beneficial health influence [20], especially their ω6 and ω3 ratio between 1.5 and 3, which is generally accepted as the balanced value for human nutrition [21]. From the results obtained in this study, it is evident that this ratio is higher due to a higher amount of ω6 fatty acids (ω6 FAs, 27.62%) compared with ω3 fatty acids (ω3 FAs, 6.09%). The prevalence of ω6 FAs over ω3 FAs is characteristic for the family Fucaceae and the genus *Fucus* [8]. Given the high content of ω6 FAs and ω3 FAs in this alga, there is a potential of using it as the source of these essential FAs. Eicosapentaenoic acid was the dominant ω3 FA with 3.71%, while arachidonic acid was found as the major ω6 FA with 15.00%, which was in accordance with the values found in *F. spiralis* [22] and *F. vesiculosus* [23]. Another study of *F. virsoides* collected from the Istrian coast of the northern Adriatic Sea [7] confirmed the dominance of oleic acid up to 33.9%, but its higher amount was found in the present study (42.28%). The difference could be due to the different collection sites (the alga in the present research was collected from the middle part of the Adriatic Sea). A previous study revealed that the content of oleic acid can also vary by the collection season, reaching the maximum in summer [24]. This trend is also noticed for arachidonic acid, while eicosapentaenoic acid content was higher during winter [25]. Generally, the brown algae are known for their dominance of oleic acid, which is confirmed for brown algae collected from different regions [20,26,27,28]. Hence, myristic acid is known as the dominant saturated fatty acid in the family Fucaceae, which was also confirmed in the present research. Najdek et al. (2014) [7] reported that *F. virsoides* contained around 14% of myristic acid, while in the current study it was present at lower abundance (10.51%).

### 2.3. Non-Targeted Screening of Less Polar Non-Volatile Compounds

FdFv sample was fractionated (Section 3.7) to obtain less polar fractions F3 and F4. To obtain the chemical composition of non-volatile less polar compounds in F3 and F4, a high-performance liquid chromatography-high-resolution mass spectrometry with electrospray ionization (HPLC-ESI(+)-HRMS) was used. The obtained chromatograms in positive ion mode contained the peaks of more than 400 individual compounds. The major compounds (in terms of signal intensity) were tentatively identified based on their elemental compositions and tandem mass spectra (Table 4). They mainly belonged to five chemical classes of natural products: fatty acid glycerides, terpenoids, steroids, carotenoids, and chlorophyll derivatives. 

Fatty acid glycerides consist of seven compounds and dominate the chromatogram. They are represented by mono- and diglycerides of stearic, palmitic, oleic, and arachidonic acids, which are found prevailing in *F. virsoides* fatty acid composition (Table 3). Three other compounds, chemically related to this group, were identified as fatty amide (docosenamide) and fatty acid esters of 2-hydroxypropanol (Rt = 15.55 and 16.55 min). It is expected that the latter two compounds and diglycerides with lower polarity and higher retention times prevail in F4, while monoglycerides are more typical for F3. 

The group of terpenoids and steroids comprises five compounds with elemental compositions C_20_H_30(32)_O_2_ and C_29_H_48_O_(2)_, respectively. Due to the diversity of isomeric compounds within this group, their reliable identification without standards is very difficult. However, the tandem mass spectrum of C_20_H_32_O_2_ demonstrating the loss of two H_2_O molecules and specific carbon backbone fragmentation pattern allows assigning the structure of diterpenoid isoamijiol previously isolated by Ochi et al. [29] from brown alga *Dictyota linearis.* Thus, another compound with a similar retention time and elemental composition, C_20_H_30_O_2_, containing only one hydroxyl, can be tentatively identified as the product of the isoamijiol oxidation possessing keto group. Three major steroids are represented by diol, monool, and the compound possessing hydroxy and keto groups (Rt = 16.25, 18.39, and 18.22 min, respectively). 

**Figure 1:**
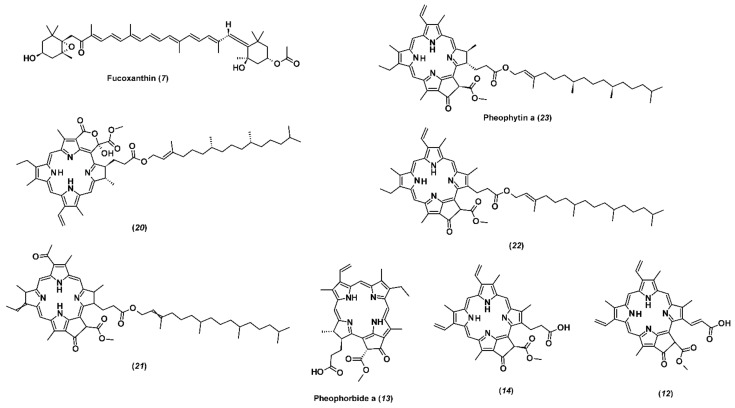
Structural formulas of identified pigments by HPLC-ESI(+)-HRMS and labelled by numbers depicted in Table 4.

Among carotenoids, only fucoxanthin (Figure 1) is included in the group of major constituents of the investigated fractions. Noteworthy is the remarkable difference in the abundance of this compound in F3 and F4. The latter contains two orders of magnitude less of this component than F3. Fucoxanthin has been the main carotenoid pigment in all the brown algae, and it is responsible for their color by masking the chlorophylls and other carotenoids [30,31]. Documented biological properties of fucoxanthin, i.e., antioxidant and anticancer [32,33], make brown algae a valuable source of this pigment. Osorio et al. [34] reported that it constituted 96% of total carotenoids from *Himanthalia elongata*, while in *Laminaria ochroleuca* and *Undaria pinnatifida* it represented 52% and 49% of the total carotenoids, respectively. Haugan and Liaaen-Jensen [35,36] reported that fucoxanthin represented up to 70% of the total carotenoids in *F. serratus* and *F. vesiculosus*. Hence, when ethanolic extracts of *F. vesiculosus* were examined, it was found that the extract which was obtained by 50–70% aqueous ethanol solution contained a higher concentration of fucoxanthin when compared with the extract obtained by 30–35% aqueous ethanol solution [37]. Another study on *F. evanescens* reported isolation and purification of fucoxanthin based on crude ethanol extract [38]. Ramus et al. [39] quantified fucoxanthin in *F. vesiculosus*, while *F. distichus* was examined for fucoxanthin presence by Terasaki et al. [40]. These differences among fucoxanthin content of different brown algae species may be due to different environmental factors and species-inherent characteristics. Maeda et al. [32] reported that low-temperature seawater and stress from the environment are important for the accumulation of fucoxanthin; in addition, higher total carotenoids content is found at colder temperatures (12 °C) [41]. *F. virsoides* used in this study was collected during winter with seawater temperature of 12 °C, which can explain high concentrations of fucoxanthin in F3. Nevertheless, a 70% fucoxanthin content decrease was observed in macroalgae exposed to high UV radiation i.e., littoral zone [39]. Matishov and Makarov [42] examined the concentrations of photosynthetic pigments (chlorophylls and carotenoids) in *F. serratus* and *F. vesiculosus* during prolonged exposure to dark and found that the amount of total carotenoids and chlorophylls was higher in the dark container. The seasonal variations and differences in geographic locations can significantly influence fucoxanthin content in macroalgae.

Chlorophyll was not detected in the investigated fractions, but its derivatives devoid of magnesium atoms constituted a large group of identified major compounds (Figure 1). The group can be divided into two subgroups, which are very different in polarity and the number of carbon atoms. The first subgroup includes four highly lipophilic compounds with elemental composition C_55_H_74(72)_N_4_O_(5-7)_ and were found in significant abundance only in F4. Its main component is pheophytin *a*, which accounts for 79% of the total signal intensity of all chlorophyll derivatives in F4 and two-thirds of the sum of both fractions (F3 + F4). It was suggested that the alcohols acidity could lead to the degradation products formation, as well as derivatives of chlorophylls [43]. The most represented derivative of chlorophyll *a* is pheophytin *a*, which is present in notable amounts in macroalgae [44]. Hence, the formation of pheophytin *a* increased the antioxidant activity of the extracts obtained from brown algae *Sargassum* spp. [45].

The other three representatives of this subgroup are pheophytin *a* derivatives, characterized by the presence of an additional double bond, carbonyl, or hydroxyl group in their composition. The second subgroup consists of three less lipophilic compounds present predominantly in F3. Their molecules contain 35 carbon, 4 nitrogen, and 5 oxygen atoms having a common structure and differing in the number of double bonds (pheophorbide *a* and its derivatives, which differ from pheophytin *a* by the absence of the long side hydrocarbon chain).

The chromatograms obtained in negative electrospray ionization mode are characterized by one order of magnitude lower signal intensities and contain significantly fewer peaks. Moreover, the presence of major compounds is observed only in F3. These include mainly glycerol molecules esterified with fatty acids and etherified with sulfonated sugar. For example, the main peak in ESI(-) chromatogram with the corresponding ion *m*/*z* 791.4965 and elemental composition C_41_H_76_O_12_S can be attributed to 3-[(9*E*)-9-octadecenoyloxy]-2-(myristoyloxy)propyl-6-deoxy-6-sulfo-α-D-glucopyranoside. In MS/MS spectrum this compound demonstrates sequential elimination of oleic and myristic acids with formation of sulfonated glucopyranose further fragmenting with the release of sulfo-anion [HSO_3_]^–^ with *m*/*z* 80.9653. However, we decided to present the results of HPLC-ESI(+)-HRMS due to the higher intensity of the peaks.

### 2.4. In Vitro Antioxidant Activity Determination

In the last decade, numerous efforts have been focused on finding important bioactive compounds with antioxidative activity from natural sources such as marine organisms [46]. Within this study, in vitro evaluation of the antioxidative activity of F3 and F4 fractions were first performed by Folin–Ciocalteu (F-C) assay. In the context of the performed research, F-C assay should not be considered as a measure of total phenolic content, but rather as the rate of overall antioxidant capacity, similar to ABTS assay [47]. Namely, many nonphenolic compounds exhibit considerable reactivity toward F-C reagent [47,48]. The study of El-Hamidi et al. [49] revealed that the interference of carotenoids in F-C reaction is quite negligible, and therefore fucoxanthin is expected not to react with F-C reagent. However, chlorophyll interacts with F-C reagent [49], resulting in an increase in the absorbance reading at 765 nm and giving a false overestimation of the concentration of phenolic compounds. Since chlorophyll derivatives were found (pheophytin *a* and its derivatives, Table 4), we can expect their contribution to the higher absorbance in F-C reaction. F-C assay showed relatively high antioxidant capacity in both fractions: F3 (75.47 ± 0.39 mg GAE/g) and F4 (55.22 ± 2.12 mg GAE/g). In comparison, other studies evaluating antioxidant activity based on total phenolic content by F-C assay of the genus *Fucus* reported the values of 80.70 and 75.96 mg/g GAE for methanolic and ethanolic extracts from *F. serratus* [50]. Total phenolic content for *F. vesiculosus* was 2.50 mg GAE/g dry weight (dw) for the extract obtained in 60% methanol [51], while a significantly higher value of 165 mg GAE/g dw was obtained in 80% ethanol extract [52]. Different results regarding polyphenolic content within genus *Fucus* indicate that the diverse chemical composition of each species is dependent on location and climate conditions, but also on the possible interaction of present nonphenolic compounds with F-C reagent. In correlation, we have also recently studied the composition and activity of another alga collected from the Adriatic Sea, red alga *Amphiroa rigida* [53]. The F-C assay showed that the antioxidant capacity of F3 and F4 fractions of *A. rigida* were 1.3- and 3.3-fold lower than in *F. virsoides*.

**Figure 2:**
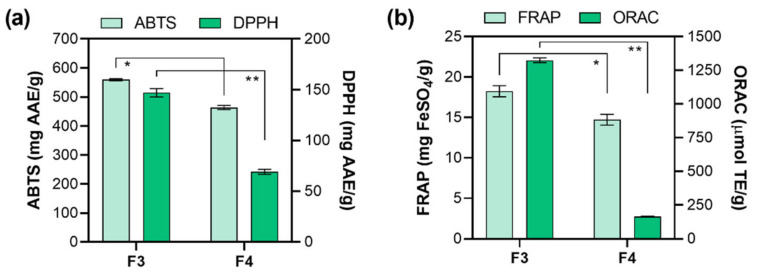
Radical scavenging effect of *F. virsoides* crude fractions using (**a**) reduction of radical cation (ABTS) and 2,2-diphenyl-1-picryl-hydrazyl-hydrate (DPPH) and (**b**) ferric reducing antioxidant power (FRAP) and oxygen radical absorbance capacity (ORAC) in vitro assays (mean ± SD; *n =* 3). An asterisk indicates a significant difference between F3 and F4 (* *p* < 0.05; ** *p* < 0.01).

The polyphenols antioxidant activity is the consequence of their ability to act as hydrogen donors, reducing agents, and free radical quenchers, or even as metal chelators [54,55]. However, pigments (i.e., carotenoids), proteins, peptides, or polysaccharides can also affect the antioxidant capacity [56]. It is necessary to apply at least two different methods for evaluating the antioxidant capacity of obtained fractions since it is known that antioxidants are usually included in several mechanisms of action [57,58]. Therefore, F3 and F4 antioxidant activity was further tested by implementing four antioxidant assays: ABTS, DPPH, ORAC (radical scavenging activity), and FRAP (ferric reducing antioxidant power). The results of ABTS and DPPH assays are presented in Figure 2a. The ABTS assay is based on absorbance inhibition (ABTS^•+^ decolorization through measuring the radical cation reduction). The results of ABTS assay showed high antioxidant activity for both fractions (F3 and F4) with the inhibition around 70% for F3 (1 mg/mL) and were presented as 559.85 ± 3.50 mg AAE/g, followed by F4 (463.70 ± 7.17 mg AAE/g). The results of DPPH assay follow the same order (F3 > F4) but differ significantly, i.e., 2.3-fold higher (*p* < 0.01) activity was observed for F3 (147 ± 4.09 mg AAE/g) than for F4 (63.68 ± 2.98 mg AAE/g). Both ABTS and DPPH assays have been frequently used in antioxidative activity evaluation because they measure the ability of reaction via both single electron transfer (SET) and hydrogen atom transfer (HAT) mechanisms. The higher antioxidant activity of methanolic fraction F3 could also be ascribed to higher fucoxanthin content (Table 4). Zaragoza et al. [37] have also reported that fucoxanthin is partly responsible for higher antioxidant activities of *F. vesiculosus* ethanolic extracts. Additionally, a lower abundance of pheophytin *a* (and its derivatives) was found in F3 (Table 4). It is known that both fucoxanthin and pheophytin *a* are the major pigments in algae and are responsible for antioxidant activity [59]. However, since the abundance of fucoxanthin (Table 4) was much higher than pheophytin *a* and its derivatives (Table 4) in F3, we can assume that it is mainly responsible for the observed antioxidant activity. ORAC assay evaluates a probe fluorescent signal that is quenched in the presence of reactive oxygen species (ROS). The antioxidant addition absorbs the ROS generated, allowing the persistence of the fluorescent signal, which is based on the antioxidant ability to quench free radicals by hydrogen donation, thus providing information on the compound mechanism action. The ORAC assay results (Figure 2b) showed that the highest antioxidant activity was also observed for F3 (1164.50 ± 27.8 μmol TE/g), while 7.2-fold (*p* < 0.01) lower activity was observed for F4 (161.933 ± 6.66 μmol TE/g), which could also be ascribed to higher abundance of fucoxanthin in F3. Lastly, FRAP assay was employed since it values the antioxidant action by single electron transfer (SET) but cannot detect the compounds acting only by radical quenching (HAT), so it can be used as a good method to estimate the antioxidant activity of different polarity fractions. Although the obtained results revealed slightly increased activity (*p* < 0.05) of F3 (18.24 ± 0.68 mg FeSO_4_/g) than F4 (14.71 ± 0.67 mg FeSO_4_/g), their similarity indicates that the present antioxidant compounds (particularly fucoxanthin in F3 and pheophytin *a*, and its derivatives, in F4) use the SET mode of action in radical quenching [60].

It can be concluded that both fractions showed higher prevention of ROS formation, with the emphasis on methanolic fraction F3 with the higher content of fucoxanthin and lower content of pheophytin *a* (and its derivatives).

### 2.5. Zebrafish Embryotoxicity

**Figure 3:**
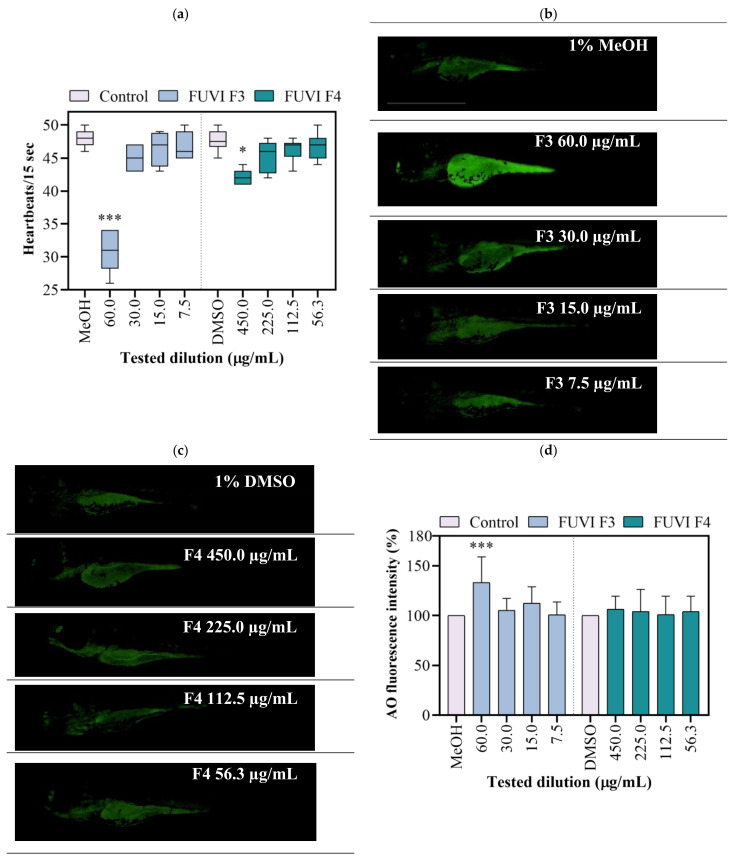
Developmental toxicity of *F. virsoides* F3 and F4 fractions in zebrafish *Danio rerio* (*n =* 30) at 96 hpf. (**a**) Heartbeat rate. A line within the box presents the median value, while the boundaries of box-plot show 25th and 75th percentiles. Above and below the box, whiskers show 10th and 90th percentiles. Representative fluorescence images of AO-stained larvae following exposure to F3 (**b**) and F4 (**c**). (**d**) The bar graph represents the AO mean fluorescent intensity in the whole larvae calculated using Image J program. An asterisk indicates a significant difference among the treatment group and negative control (* *p* < 0.05; *** *p* < 0.001).

The exposure to tested F4 fraction dilutions had no statistically significant impact on survival, incidence of developmental abnormalities, and hatching success. Contrary, the exposure to F3 fraction resulted in a concentration-dependent effect, causing 100% of mortality on 120 µg/mL. Although 60 µg/mL induced no mortality, the occurrence of developmental abnormalities was recorded among 55.2 ± 5.0% of survived larvae (data not shown). F3 dilutions ≤ 30 µgm/L showed no negative effect during 96 h of zebrafish ontogenesis. Control treatment groups developed normally with a mortality rate ≤5%. Within this study, zebrafish were also used to evaluate the cardiotoxicity of F3 and F4. As shown in Figure 3a, the highest tested concentrations of F3 (60.0 µgm/L) and F4 (450.0 µg/mL) significantly decreased heartbeat frequency by 35.9% (*p* < 0.001) and 12.1% (*p* < 0.05), respectively, compared with the respective negative control groups. As an additional toxicity parameter, the cell death was determined in live larvae using acridine orange staining assay. Exposure to 60.0 µg/mL of F3 significantly increased the mean green fluorescence intensity (for 33.3%; *p* ≤ 0.001; Figure 3b,d). Other treatments (all tested F4 dilutions and ≤30 µg/mL of F3) exerted no significant cytotoxic effect in zebrafish larvae (Figure 3c,d). In accordance with the obtained results, 30.0, 15.0, and 7.5 µg/mL of F3 and 450.0, 225.0, and 112.5 µg/mL of F4 were selected for further experimentation.

### 2.6. In Vivo Antioxidant Activity (Zebrafish Model)

**Figure 4:**
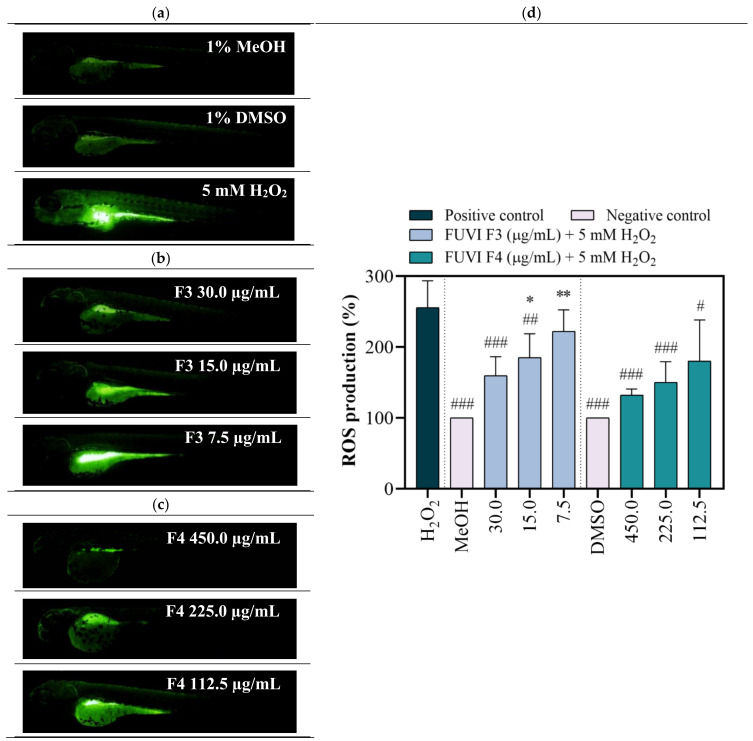
In vivo determination of the antioxidant potential of *F. virsoides* F3 and F4 fractions. Representative fluorescence images of DCF-stained larvae following exposure to (**a**) controls (1% MeOH, 1% DMSO, 5 mM H_2_O_2_), (**b**) F3 + 5 mM H_2_O_2_, and (**c**) F4 + 5 mM H_2_O_2_. Scale bar = 100 µm. (**d**) The bar graph shows DCF mean fluorescent intensity in the whole larvae calculated using Image J program. An asterisk indicates significance compared with representative negative control, while # represents significance compared with H_2_O_2_ treated group (*/# *p* < 0.05; **/## *p* < 0.01; ***/### *p* < 0.001).

Zebrafish as a model organism is widely used in (eco)toxicity studies, drug discovery, and developmental biology due to its numerous beneficial features, including rapid development, high fecundity, optical transparency during the whole embryonic development, but also, most importantly, biological similarities with mammalian physiological pathways and functional domains of disease-associated genes [61]. Recently, the zebrafish model stimulated with H_2_O_2_ was successfully implemented in numerous in vivo experiments that investigated the antioxidant effect of natural bioactive molecules from various marine organisms (*Padina boryana* [62], *Undaria pinnatifida* [63], *Ecklonia cava* [64], *Hizikia fusiforme* [65], etc.). Regardless, to the best of our knowledge, this study is the first that comprehensively assessed the toxicity and antioxidant activity of *F. virsoides* fractions. Within the study, after the evaluation of radical scavenging in vitro, the zebrafish embryo model was employed to confirm the protective effects of *F. virsoides* fractions on H_2_O_2_-induced ROS generation in vivo. For this purpose, zebrafish embryos were exposed to F3 and F4 fractions in the presence and absence of H_2_O_2_. The H_2_O_2_ treated group demonstrated significantly high DCF fluorescence intensity compared with negative control groups (MeOH, DMSO), indicating high ROS generation (Figure 4a). F3 and F4 treatments significantly downregulated the fluorescence intensity in the H_2_O_2_-treated groups in a concentration-dependent manner (Figure 4b,c). F3 fraction at 30 µg/mL and 15 µg/mL significantly decreased the ROS formation in H_2_O_2_-treated zebrafish by 37.8% (*p* ≤ 0.001) and 27.4% (*p* ≤ 0.01), respectively. Pretreatment with F4 fraction induced a similar, but more pronounced effect (Figure 4d). All three tested concentrations of F4 fraction significantly decreased H_2_O_2_-induced ROS levels for 48.2% (*p* ≤ 0.001; 450.0 µg/mL), 41.2% (*p* ≤ 0.001; 225.0 µg/mL), and 29.5% (*p* ≤ 0.05; 112.5 µg/mL). The ROS formation in negative control groups was considered to be 100%. The obtained results show that both F3 and F4 fractions exerted a protective effect against H_2_O_2_-induced oxidative stress during zebrafish development. Although the obtained results seem to differ from the in vitro measurements (ABTS, DPPH, FRAP, and ORAC) that confirmed the higher antioxidant activity of F3, one should notice that the difference might be associated with the higher concentration range tested (450.0–112.5 µg/mL of F4) when compared with F3 (30.0–7.5 µg/mL). As reported above, the concentration range of F3 fraction was lowered due to the recorded toxicity at concentrations ≥60 µg/mL. Nonetheless, one can notice that 150.3 µg/mL of F4 fraction exerted a similar protection from ROS production in H_2_O_2_-treated zebrafish (decrease of fluorescence intensity of 41.2%) as 30 µg/mL of F3 fraction (decrease of 37.5%). Considering the potential further application, F3 appeared to be a better choice due to observed antioxidant potential with lower applied concentration. The chemical characterization of *F. virsoides* F3 (Table 4) revealed the presence of fucoxanthin, which already demonstrated radical scavenging and singlet oxygen quenching abilities [66]. Fucoxanthin demonstrated significant protection against H_2_O_2_ in kidney epithetical (Vero) [67], human umbilical vein endothelial (HUVEC) [68], rat pheochromocytoma (PC-12) [69], and neuroblastoma (SH-Sy5Y) [70] cell lines. Kang et al. [68] showed that fucoxanthin isolated from brown alga *Ishige okamurae* had a protective effect against glucose-induced oxidative stress in a zebrafish model, i.e., at 25, 50, and 100 µM significantly reduced ROS generation for 24%, 35%, and 57%, respectively. Fucoxanthin concentration may vary depending on the species of brown algae, abiotic factors, etc., which reflects in the ability of fucoxanthin to increase cell viability compromised by oxidative stress inductor from 50% to almost 90% [69]. Additionally, pheophytin *a* played a significant role in elevating antioxidant activity, thus enabling protection during co-exposure with H_2_O_2_. As recently reported by Yalçın et al. [59], pheophytin *a* was one of the main pigments that increased the antioxidant activity of the raw extract of green, brown, and red algae. Lanfer-Marquez et al. [71] evidenced the high antioxidative potential of pheophytin *a* (~70% between 50 and 100 ppm BHT equivalents). Considering its higher abundance in F4 fraction, we can assume that pheophytin *a* was one of the compounds responsible for beneficial properties observed within this study. It is important to emphasize that observed antioxidant activity of the fractions is not caused by individual compound, so possible interaction between bioactive compounds and their synergy in such a complex mixture should be considered.

Collectively, results obtained with in vitro and in vivo antioxidant assays confirm that *F. virsoides* exhibits strong antioxidant activity potential that suppresses ROS generation. These findings emphasize the potential of *F. virsoides*, thus shedding a light on its future practical implementation.

### 2.7. Antimicrobial Activity

Using the disk diffusion method, F3 and F4 did not exhibit antimicrobial activity against any of the bacterial and fungal indicator species. There are several reports of antimicrobial activity of macroalgae of the *Fucus* genus with somewhat conflicting results that may be due to algal and indicator species used, location of the study, a season of the year, extraction procedures, and amount of material and method of antimicrobial testing performed. Two related studies of *F. virsoides* have been reported from the Adriatic Sea region. One study sampled the alga from the Venice lagoon, Italy, in March and performed ethanolic and aqueous extractions directed for polysaccharides. In that study, the disks were loaded with 2 mg of extracts, and mild inhibitions (8–13 mm) were observed against 5 out of 7 bacterial aquaculture pathogens and, of human pathogens, against one *Salmonella* spp., but no inhibition of *P. aeruginosa* and *S. aureus* [72]. The authors further reported the antibacterial activities of sulphated polysaccharides from the aqueous extracts as being stronger than the ethanolic ones. The other study was performed in the Bay of Kotor, Montenegro, and compared seasonal variation in biological activities between summer and fall seasons [8]. That study also performed extraction using MeOH:DCM as in this study, but the fractions were obtained using *n*-butanol, ethyl-acetate, and petroleum-ether and minimum inhibitory concentrations (MIC) ranged from 0.31 to 11.72 mg/mL for five bacteria (including all species except *P. aeruginosa* from this study) and 5 fungi (including *C. albicans*). The authors reported ethyl-acetate fraction to exert the strongest effect (both antibacterial and antifungal) that was not affected by the season of sampling since MICs were the same or within one 2-fold dilution. The MIC values of crude extracts were similar between seasons for the vast majority of indicator species. In contrast, the other two fractions differed largely in the observed MICs between the two seasons. Further studies are needed to explain the reasons for these differences.

## 3. Materials and Methods

### 3.1. Chemicals

The fibres for HS-SPME containing DVB/CAR/PDMS (Divinylbenzene/Carboxen/Polydimethylsiloxane) or PDMS/DVB (Polydimethylsiloxane/Divinylbenzene) were obtained from Supelco Co. (Bellefonte, PA, USA). The fatty acids methyl esters (FAMEs) used for the determination of fatty acids were purchased from Supelco Co. (Bellefonte, PA, USA). Mueller–Hinton and YPD agars, antimicrobial agent norfloxacin, chloramphenicol, the standards of gallic acid (>97.5%), ABTS (diammonium salt of 2,2′-azino-bis(3-ethylbenzthiazolin-6-yl)sulfonic acid, >99.0%), DPPH (2,2-diphenyl-1-picrylhydrazyl), TPTZ (2,4,6-tripyridyl-S-triazine, ≥98%), Trolox solution (6-hydro-2,5,7,8- tetramethylchroman-2-carboxylic acid, 97%), AAPH (2,2-azobis (2-methylpropionamidine) dihydrochloride, 97%), fluorescent dies acridine orange, 2′,7′-dichlorofluorescein (DCF, ~90%), and dichloro-dihydro-fluorescein diacetate (DCF-DA) were purchased from Sigma-Aldrich (St. Louis, MO, USA).

Suspension medium was purchased from bioMerieux (Lyon, France), YPD broth was used from Sigma-Aldrich (St. Louis, MO, USA), Mueller Hinton agar was obtained from Merck (Darmstadt, Germany), and bacteriological and tryptic soy agars were purchased from Oxoid (Basingstoke, UK). Nystatin was obtained from Acros Organics (Geel, Belgium) and chloramphenicol and norfloxacin from Sigma-Aldrich. Methanol (p.a.), ethanol (p.a.), dimethyl sulfoxide (DMSO, p.a.), hydrochloric acid (HCl, p.a.), iron (III) chloride (FeCl_3_, p.a.), Folin–Ciocalteu reagent and NaHCO_3_ (p.a.) were obtained from Kemika (Zagreb, Croatia), while potassium persulfate (>98%) was purchased from Scharlau (Germany). Hydrogen peroxide (H_2_O_2_, 30%) was obtained from Alkaloid Skopje (Macedonia).

HPLC gradient grade acetonitrile obtained from Khimmed (Moscow, Russia), HPLC grade formic acid was purchased from Sigma-Aldrich (St. Louis, MO, USA), and ultrapure (Type I) Milli-Q water were used for mobile phase preparation in HPLC-ESI-HRMS analyses.

Zebrafish *D. rerio* adults of wild-type WIK strain were obtained from the European Zebrafish Resource Center of the Karlsruhe Institute of Technology (KIT).

Used solvents were of HPLC grade and were obtained from J.T. Baker (New Jersey, PA, USA). The standard compounds used in Table 1 and Table 2 were purchased from Sigma-Aldrich (St. Louis, MO, USA).

### 3.2. The Sample Collection and Preparation Procedure

*Fucus virsoides* J. Agardh, 1868, was gathered in the middle part of the Adriatic Sea coast, Novigrad sea area, in February 2021 (44°12′02″ N; 15°28′51″ E). Single point sample collection enabled a representative sample. *F. virsoides* was collected from a depth of 0.5 m at the sea temperature of 12 °C. The sample was collected and placed in an airtight plastic bag with surrounding seawater and was immediately transported to the laboratory.

Before HS-SPME and HD, the sample was held at 4 °C in the dark and the extraction and HD were conducted within 48 h of the sampling. The sample of *F. virsoides* was cut into small pieces, and the excess seawater was removed by the filter paper layers as was performed in the previous investigations [15,53,73]. A part of collected *F. virsoides* was air-dried 14 days at a room temperature in the dark and used as air-dried material for HD and HS-SPME.

For the extraction of fatty acids and less polar non-volatiles (the procedures explained in the Section 3.6, Section 3.7, Section 3.8, Section 3.9, Section 3.10, Section 3.11 and Section 3.12)*,* fresh *F. virsoides* was freeze-dried. Before the freeze-drying, the sample was washed five times in water and twice in deionized water, then it was cut in slices (5–10 mm) and frozen at −60 °C for 24 h in an ultra-low freezer. Five trays of frozen samples were placed in a laboratory freeze dryer (CoolSafe PRO, Labogene, Denmark). The freeze-drying was performed under a high vacuum (0.13–0.55 hPa) for 24 h with −30 °C and 20 °C as the primary and secondary drying temperatures. Freeze-dried samples were further used for the analysis of fatty acids and less polar non-volatiles and for the antioxidant and antimicrobial activities testing.

### 3.3. Headspace Solid-Phase Microextraction (HS-SPME)

HS-SPME was conducted with a manual SPME holder using two fibers containing divinylbenzene/carboxen/polydimethylsiloxane (DVB/CAR/PDMS) or polydimethylsiloxane/divinylbenzene (PDMS/DVB). The fibers were conditioned according to Supelco Co instructions. Cut samples (1 g) were set separately in glass vials (5 mL) and sealed hermetically using PTFE/silicone septa. The vials were placed in a water bath (60 °C) during equilibration (15 min) and the extraction by HS-SPME (45 min). After the extraction, the SPME fiber was withdrawn, taken away from the vial, and inserted into the GC-FID and GC-MS injector (250 °C) for the thermal desorption (6 min). The treatment was similar to previous research [15,53,73]. HS-SPME was performed in triplicate.

### 3.4. Hydrodistillation (HD)

A modified Clevenger apparatus was used for HD (2 h). The solvent trap was applied (1 mL of pentane: diethyl ether (1:2 *v*/*v*)). The fresh and air-dried *F. virsoides* (15 g; cut into little pieces) were used for HD. HD was performed in triplicate for fresh and air-dried sample. The volatile oil dissolved in the trap was removed with a pipette, dried over MgSO_4_ layer, and slowly concentrated by the slow nitrogen flow until 0.2 mL. The volume of 2 μL was used for GC-FID and GC-MS analyses.

### 3.5. Gas Chromatography and Mass Spectrometry (GC-FID/MS) Analysis

GC-MS analyses were performed on a gas chromatograph model 7890A (Agilent Technologies, Palo Alto, CA, USA) equipped with an HP-5MS capillary column (5% phenyl-methylpolysiloxane, Agilent J and W; 30 m × 0.25 mm i.d., coating thickness 0.25 µm) and a flame ionization detector (FID). The GC conditions were described previously [15,53,73]. The carrier gas was helium (He 1.0 mL/min). The oven temperature was set up at 70 °C for 2 min, then the temperature was increased from 70 °C to 200 °C at a rate of 3 °C/min, and held at 200 °C for 15 min. The GC-MS analyses were performed on a gas chromatograph model 7820A (Agilent Technologies, Palo Alto, CA, USA) equipped with a mass selective detector (MSD) model 5977E (Agilent Technologies, Palo Alto, CA, USA) and the same HP-5MS capillary column applying the same conditions as for the GC-FID analysis. The MSD (EI mode) was used at 70 eV, and 30–300 amu mass range was applied.

The compounds identification was based on retention indices (RIs) determined relative to *n*-alkanes (C_9_–C_25_) retention times and their comparison with data in the literature (National Institute of Standards and Technology), as well as by their mass spectra compared with the spectra from Wiley 9 (Wiley, New York, NY, USA) and NIST 17 (D-Gaithersburg) mass spectral libraries. The identification of the majority of the compounds from Table 1 and Table 2 was confirmed by co-injection with the authentic standard compounds. The percentage composition was calculated using the normalization method (without correction factors). The average component percentages in Table 1 and Table 2 were calculated from three replicate of GC-FID and GC-MS analyses.

### 3.6. The Analysis of Fatty Acids by GC-FID

The fatty acid methyl esters were prepared according to HRN EN ISO 12966-2:2017 standard [74]. They were analyzed by gas chromatography (GC) with FID according to HRN EN ISO 12966-4:2015 [75]. Agilent Technologies gas chromatograph 7890A (Lake Forest, CA, USA) with ZB-WAX capillary column (Phenomenex, Torrance, CA, USA; 25 m x 0.25 mm i.d. the stationary phase thickness 0.25 µm) and a split–splitless injector (260 °C), and FID (280 °C) was used. The sample volume of 5 µL was injected with a split ratio of 1:40. Starting column temperature was 60 °C with 2 min holding time. The oven temperature was increased at the rate of 13 °C/min to 150 °C, then was heated to 240 °C at the rate of 2 °C/min. Helium (He; 99.9999%) was the carrier gas at a flow rate of 3 mL/min. The hydrogen flow was 70 mL/min, air flow was 450 mL/min, and the makeup gas flow (nitrogen) was 15 mL/min.

In Table 3 37 fatty acid methyl ester standard compounds were used for the identification of obtained fatty acid methyl esters (by comparison with the retention times at the same operating conditions). The results (Table 3) are expressed as % of individual fatty acids to total fatty acids. The method detection limit was 0.1%.

### 3.7. Fractionation Using Solid-Phase Extraction (SPE)

The freeze-dried sample of *F. virsoides* (FdFv) was extracted (the solvent:solid ratio was 10 mL/g) three times for 5 min with sonication (ultrasound-bath Elma, Elmasonic P 70 H, Singen, Germany; 37 kHz/50 W) applying solvents mixture methanol:dichloromethane (MeOH/DCM, 1:1, *v*/*v*). The obtained extract was evaporated under nitrogen (5.0, Messer, Croatia) to remove the solvent, and it was mixed with C18 powder (Macherey-Nagel Polygoprep 60-50 C18, Fisher Scientific, Massachusetts, USA; 40–63 µm). The obtained dry extract was then placed on an SPE cartridge (C18, bed weight 1g, column capacity 6 mL, particle size 40 µm; Agilent Bond Elut, Waldbronn, Germany) which was conditioned prior to the fractionation with methanol and ultrapure water. Then the sample was eluted by applying solvents of decreasing polarity to obtain the fractions F1 to F4: F1 (with H_2_O), F2 (with H_2_O/MeOH (1:1, *v*/*v*)), F3 (with MeOH), and F4 (with MeOH/DCM (1:1, *v*/*v*)). The water-soluble components were eluted in F1 and F2 and enabled purification of the targeted less-polar compounds in F3 and F4. The obtained fractions F3 and F4 were dried by SpeedVac (SPD1030, Thermo Scientific, Waltham, MA, USA) and stored at 4 °C in dark. The fractionation with SPE was performed in triplicates.

### 3.8. Non-Targeted Screening by High Performance Liquid Chromatography-High-Resolution Mass Spectrometry (HPLC-ESI-HRMS)

HPLC-HRMS analyses were performed using LC-30 Nexera chromatograph (Shimadzu, Kyoto, Japan) equipped with a vacuum degasser, two chromatographic pumps LC-30AD, autosampler SIL-30AC, column thermostat STO-30, combined with quadrupole-time-of-flight (Q-TOF) mass spectrometer TripleTOF 5600+ (AB Sciex, Concord, Canada) with Duospray ion source.

The chromatographic separations were achieved using a Nucleodur PFP column (Macherey-Nagel, Duren, Germany; 150 × 2 mm, particle size 1.8 µm with pentafluorophenyl-propyl stationary phase) at 40 °C. For the mobile phase, a mixture of water (A) and acetonitrile (B) (both containing 0.1% formic acid) was used. The gradient program was applied as follows: 0–1 min (30% B), 1–22 min (B linear gradient to 100%), 22–25 min (100% B). The flow rate of mobile phase was 0.25 mL/min, and the injection volume was 5 µL.

Mass spectrometry detection was performed using positive and negative electrospray ionization (ESI+ and ESI−). Tandem (MS/MS) mass spectra were recorded using collision-induced dissociation (CID) in information-dependent acquisition (IDA) mode for precursor ions with the signal intensities above 200 cps threshold. The maximum number of precursor ions simultaneously subjected to CID was 15. The ion source parameters were: nebulizing and drying gas (air) pressure 40 psi, curtain gas (nitrogen) pressure 30 psi, ESI capillary voltage 5.5 kV and 4.5 kV for positive and negative ion modes, respectively, and the source temperature 300 °C. The recording mass spectra parameters were: declustering potential 80 V, *m*/*z* range 100–1000 (MS) and 20–1000 (MS/MS) and acquisition time 150 ms. Nitrogen was applied as the collision gas (collision energy was 40 eV with a spread of 20 eV). The mass scale calibrations (in the MS and MS/MS modes) were completed prior to each run in an automatic regime using a sodium formate solution as the standard.

The data processing was performed by PeakView, MasterView, and Formula Finder (AB Sciex, Concord, ON, Canada) software packages. The elemental compositions of the compounds were determined based on the accurate masses of the corresponding protonated or deprotonated molecules, their isotopic distributions, and the product ions *m*/*z* in MS/MS spectra. The tentative identification of detected components was carried out on the basis of their elemental compositions, tandem mass spectra, and automatic search in the ChemSpider database, with a further selection of hits matching with MS/MS data.

### 3.9. In Vitro Antioxidant Activity Determination

The dry residues of F3 and F4 were dissolved in a proper solvent (methanol for F3 and dimethyl sulfoxide (DMSO) for F4). F3 and F4 were prepared at a maximum concentration of 10 mg/mL (the stock concentration was highly dependent on the obtained fractions mass). Afterward, appropriate dilutions of each sample were prepared.

All measurements were performed on a spectrophotofluorimeter microplate reader (Infinite M200 PRO, TECAN, Mannedorf, Switzerland) in the multi-well plate (96-well) in triplicates.

The Folin–Ciocalteu method was used [76] with some adaptations. In brief, 100 μL of the fraction was mixed with 750 μL of Folin–Ciocalteu reagent (previously 10-fold diluted with distilled water) and allowed to stand for 5 min at room temperature. Afterward, 750 μL of sodium bicarbonate solution (60 g/L) was added to the mixture. After incubation at room temperature for 90 min, the absorbance at 750 nm was read. Folin–Ciocalteau assay was calibrated against gallic acid standard, and the results are expressed as mg gallic acid equivalent (GAE) per g of the fraction.

The antioxidant capacity was determined by four assays (ABTS, DPPH, FRAP, and ORAC). Ascorbic acid was used as the reference for both DPPH and ABTS assays, and the results are expressed as milligram ascorbic acid equivalents per gram of the sample (mg/g AAE). DPPH radical scavenging assay [77] was adapted to microscale with slight modifications. The volume of 25 μL of the prepared diluted fraction was mixed with 200 μL of methanol and prepared DPPH reagent in methanol (240 μg/mL) in a 96-well plate. The reaction mixture was kept in the dark for 30 min, after which the absorbance of the solution was measured at 490 nm. Appropriate blanks (methanol/DMSO) and standards (ascorbic acid solution) were run simultaneously. The ABTS^+•^ radical-cation discoloration assay was measured by spectroscopy at 734 nm [78] and adapted to microscale with slight modifications. The ABTS^+•^ stock solution was prepared from a 7 mM ABTS with 2.45 mM of potassium persulfate dissolved in 5 mL of distilled water and allowed to react at room temperature in the absence of light for 17 h. The working solution of the already preformed ABTS^+•^ radical-cation was diluted with ethanol to achieve an absorbance of 0.700 ± 0.02. The reaction mixture consisted of the sample and ABTS^+•^ solution, resulting in inhibition between 20% and 80% (the blank was represented by the used solvent for each fraction).

The FRAP assay was performed according to the method of Benzie and Strain [79] with minor modification. In brief, FRAP reagent was freshly prepared by mixing equal volumes of a 10 mM TPTZ solution in 40 mM HCl and an aqueous 20 mM FeCl_3_ solution and diluting the mixture five times in 0.25 M acetate buffer (pH 3.6), followed by heating to 37 °C. Next, 100 μL of the sample fraction or Trolox solution, which was used as a positive control, was mixed with 3.9 mL of FRAP reagent, and the absorbance was determined at 593 nm after the incubation at 37 °C for 10 min. The results are expressed as mg FeSO_4_/g fraction.

The oxygen radical absorbance capacity (ORAC assay) was determined as described by Huang and colleagues [80], with minor modification. Different concentrations of each fraction were prepared with the corresponding solvent (methanol for F3 and DMSO for F4). A defined volume of 25 μL of diluted samples was added in black 96-well flat-bottom plates. The same quantity of Trolox (6.25–100 μM) and solvents were applied in the plates as standard and blanks, respectively. Afterward, 150 μL DCF solution (1:500, *v*/*v* in 25 mL 75 mM PBS) was added, and the mixture was incubated 30 min at 37 °C in the shaking incubator (New Brunswick, Innova 42). Then, to start the reaction, 25 μL AAPH was added to the mixture, and the fluorescence was recorded for 16 h every 10 min (overnight kinetic cycle). The samples were measured at 485 nm excitation wavelength and 528 nm emission wavelength of with an optimal fluorescence gain of 188. The measured ORAC is expressed as μmol Trolox equivalents (TE)/g of the fraction.

### 3.10. Zebrafish Embryotoxicity Test

Zebrafish maintenance and embryo production are described in detail in our previous research [81].

In order to determine potential toxicity and to establish the concentration range of interest, zebrafish embryos (4- to 64-blastomeres) were exposed to a successive dilution of the fractions F3 (120–7.5 µg/mL) and F4 (450–56.3 µg/mL) following OECD 203 Guideline [82]. Final MeOH and DMSO concentrations within the tested samples did not exceed 1% [83]. The test was conducted in 24-well plates. Two embryos were exposed per well containing 1 mL of the tested sample, amounting to a total of 30 specimens per treatment. During 96 h of development, the specimens were incubated at 27.5 ± 0.5 °C (Innova 42 incubator shaker; New Brunswick, Canada).

At 96 h of exposure, mortality and developmental abnormalities were recorded using an inverted microscope (Olympus CKX41) equipped with Leica EC3 digital camera and LAS EZ 3.2.0 digitizing software. Moreover, cardiotoxicity was determined at 96 hpf by direct visual observation of zebrafish cardiac ventricles per 15 sec. Hatching was observed and was taken as the rupture of the chorion enabling the release of larvae. Cell death was detected in larvae at 96 hpf by staining with acridine orange (AO), a nucleic acid-selective dye that interacts with DNA and RNA, following the previously reported method [84].

### 3.11. In Vivo Antioxidant Activity Determination by Zebrafish Model

The protective response of the fractions F3 and F4 against H_2_O_2_-induced toxicity was investigated according to Wang et al. [14]. Considering the results obtained within the embryotoxicity test, zebrafish embryos (*N* = 30) at 4 hpf were treated with 30.0, 15.0, and 7.5 µg/mL of F3 and 450.0, 225.0, and 112.5 µg/mL of F4. After 4 h of pretreatment, 5 mM of H_2_O_2_ was added to the medium. Negative control groups were exposed to 1% MeOH and 1% DMSO, while positive control group was treated with 5 mM of H_2_O_2_. After 96 h of exposure, larvae were observed using a Leica DMIL LED inverted microscope equipped with an EC3 digital camera.

In order to visualize and quantify the amount of oxidative stress, larvae were treated with 10 µM of fluorogenic dye dichloro-dihydro-fluorescein diacetate (DCFDA), which diffuses into the cells. Subsequently, it hydrolyzes by intracellular esterases to a non-fluorescent compound, which is then oxidized by ROS into 2′,7′–dichlorofluorescein (DCF) [14,85]. Such a reaction product is detectable by fluorescence spectroscopy. DCFDA staining was performed following the previously described method [84]. Stained larvae were photographed using a fluorescent microscope (Olympus^®^ BX51 light binocular microscope fit up with the Microsoft^®^ AnalySIS Soft Imaging System Software) with a green fluorescent filter. The fluorescence intensity of images was quantified using ImageJ software. The antioxidant potential of the fractions should be evident in a decrease of fluorescence intensity in the specimens exposed to a mixture of individual fraction and H_2_O_2_, compared with individuals exposed to H_2_O_2_.

Data obtained from in vitro and in vivo antioxidant testing were statistically analyzed using GraphPad Prism 6.0 (GraphPad Software Inc., San Diego, CA, USA). The results are expressed as means ± SD, and *p* < 0.05 was used as a cut-off value of statistical significance throughout the manuscript. One-way analysis of variance (ANOVA) and Tukey’s post hoc test were performed to examine the significance of the difference between treatments.

### 3.12. Testing of Antimicrobial Activity

The fractions F3 and F4 were tested according to CLSI guidelines for antimicrobial activity using disk diffusion method [86,87] with minor modifications. The stock solutions were prepared at 20 mg/mL (*w*/*v*) for F4 in DMSO and 10 mg/mL (*w*/*v*) for F3 in ethanol, with an aliquot of F3 also dissolved in DMSO. An amount of 30 mL of these solutions and their dilutions with distilled water were loaded on 6 mm sterile disks, resulting in amounts ranging from 86 to 600 µg per disk. Fresh overnight growth of bacteria at 37 °C on tryptic soy agar was used to prepare inocula to 0.5 MacFarland by turbidity adjustment in sterile 5 mL 0.85% suspension medium and using sterile cotton swabs subsequently plated on Mueller–Hinton agar for bacteria and YPD agar (from YPD broth), supplemented with bacteriological agar (1.5% (*w*/*v*)) for fungi. The diameter of the zones of inhibition was read and are expressed to the nearest millimeter. All tests were conducted in technical duplicates on two independent occasions. Each experimental run comprised the solvent control and antimicrobial agents chloramphenicol or norfloxacin for bacteria and nystatin for fungi. The bacterial panel consisted of Gram-positive (*Bacillus subtilis* subsp. *spizizenii* ATCC 6633 and *Staphylococcus aureus* ATCC 6538), Gram-negative (*Pseudomonas aeruginosa* NCTC 12903 and *Escherichia coli* NCTC 12241), and indicator species and the fungal panel of *Candida albicans* ATCC 90028 yeast. All tests were conducted at 35 °C in aerobic atmosphere.

## 4. Conclusions

The volatile profiles revealed a significant influence of drying when the headspace and hydrodistillate were compared. The major HS-FrFv compound was pentadecane, followed by pentadec-1-ene, while in HS-DrFv, pentadec-1-ene was not present, indicating occurrence of oxidation and decomposition reactions during drying. The oxidation products were also identified in HD-DrFv with tridecanal as the major compound, along with unsaturated aldehydes whose percentages were higher than in HD-FrFv. Even though fatty acids were the minor constituents in HD-FrFv, they were detected in *F. virsoides* after derivatization, and the results show that the main fatty acids were oleic acid, as the dominant one, followed by arachidonic acid and myristic acid. It was suggested that some volatile compounds were formed from the cleavage of the double bonds of corresponding fatty acids. According to the high ω6 and ω3 fatty acids content, *F. virsoides* can be considered as the source of essential fatty acids. The major less polar non-volatiles were identified by HPLC-ESI(+)-HRMS, including five chemical classes of natural products: fatty acid glycerides, terpenoids, steroids, carotenoids, and chlorophyll derivatives. The major ones were fucoxantin, pheophytin *a*, and its derivatives.

When the antioxidant activity was investigated by implementation of four antioxidant assays—ABTS, DPPH, ORAC, and FRAP—the results revealed that the methanolic fraction of *F. virsoides* F3 showed higher prevention of ROS formation compared with F4. Similar results were obtained when the zebrafish embryo model was employed and confirm the protective effects of *F. virsoides* fractions on H_2_O_2_-induced ROS generation in vivo. Fucoxanthin was the dominant pigment found in F3 that can be related to the higher antioxidant activity of that fraction, while the activity of F4 can be connected with pheophytin *a* and its derivatives.

Such findings point out *F. virsoides* as an inexhaustible source of bioactive compounds with desirable bioactivities.

## Figures and Tables

**Table 1:** The headspace VOCs of *F. virsoides* investigated by HS-SPME after GC-FID/MS analyses.

No.	Compound	RI	A Av ± SD	B Av ± SD	C Av ± SD	D Av ± SD
1.	Hexanal ^S^	<900	-	1.14 ± 0.03	-	0.72 ± 0.01
2.	3-Methylbutanoic acid ^S^	<900	-	0.25 ± 0.02	-	-
3.	Heptanal ^S^	901	-	1.41 ± 0.02	-	0.60 ± 0.02
4.	(*E*)-Hept-2-enal	962	-	0.35 ± 0.01	-	-
5.	Benzaldehyde ^S^	968	-	1.43 ± 0.04	-	1.18 ± 0.03
6.	Oct-1-en-3-one ^S^	983	-	0.18 ± 0.01	-	0.14 ± 0.01
7.	Oct-1-en-3-ol ^S^	983	-	0.87 ± 0.02	-	0.52 ± 0.01
8.	6-Methylhept-5-en-2-one ^S^	988	-	0.47 ± 0.02	-	-
9.	Octanal ^S^	1003	-	0.56 ± 0.01	-	-
10.	Benzyl alcohol ^S^	1044	-	19.67 ± 0.42	-	15.75 ± 0.51
11.	Nonanal ^S^	1106	-	3.36 ± 0.05	-	1.98 ± 0.02
12.	Tridecane ^S^	1300	-	0.89 ± 0.01	-	1.66 ± 0.02
13.	Pentadec-1-ene ^S^	1493	17.98 ± 0.82	-	11.00 ± 0.50	-
14.	Pentadecane ^S^	1500	61.90 ± 1.51	60.27 ± 2.00	71.55 ± 1.98	71.43 ± 1.84
15.	Tridecanal ^S^	1511	-	1.13 ± 0.02	5.45 ± 0.10	1.03 ± 0.03
16.	(*E*)-Pentadec-7-ene	1517	9.12 ± 0.80	-	-	-

A-fresh sample investigated by the fiber DVB/CAR/PDMS; B-fresh sample investigated by the fiber PDMS/DVB; C-air-dried sample investigated by the fiber DVB/CAR/PDMS; D-air-dried sample investigated by the fiber PDMS/DVB; RI-retention indices determined relative to the alkanes C_9_–C_25_; Av-average area percentage determined by GC-FID/MS of 3 replicates, SD-the area percentages standard deviation for 3 replicates; ^S^-identification confirmed by standard compound; - the compound was not detected.

**Table 2:** The compositions of *F. virsoides* volatile oil investigated by HD after GC-FID/MS analyses.

No.	Compound	RI	E Av ± SD	F Av ± SD
1.	(*E*)-Hex-2-enal ^S^	<900	-	1.13 ± 0.03
2.	Heptanal ^S^	901	-	0.86 ± 0.05
3.	(*Z*)-Hept-2-enal ^S^	961	0.32 ± 0.01	0.42 ± 0.02
4.	Benzaldehyde ^S^	968	0.09 ± 0.01	0.69 ± 0.02
5.	Oct-1-en-3-ol ^S^	983	-	0.73 ± 0.03
6.	Octan-2,3-dione ^S^	985	-	0.24 ± 0.01
7.	2-Pentylfuran ^S^	993	0.15 ± 0.01	0.70 ± 0.02
8.	(*E,E*)-Hepta-2,4-dienal ^S^	999	-	0.28 ± 0.01
9.	Octanal ^S^	1003	-	0.23 ± 0.01
10.	(*E,Z*)-Hepta-2,4-dienal	1007	-	0.13 ± 0.01
11.	(*Z,Z*)-Hepta-2,4-dienal ^S^	1015	-	0.63 ± 0.05
12.	2,2,6-Trimethylcyclohexanone ^S^	1040	0.01 ± 0.01	0.22 ± 0.03
13.	Benzyl alcohol ^S^	1044	0.62 ± 0.05	0.67 ± 0.05
14.	Phenylacetaldehyde ^S^	1050	0.54 ± 0.03	0.87 ± 0.02
15.	(*E*)-Oct-2-enal ^S^	1062	-	0.45 ± 0.02
16.	(*E*)-Oct-2-en-1-ol ^S^	1073	-	0.32 ± 0.02
17.	(*E,E*)-Octa-3,5-dien-2-one	1074	-	0.44 ± 0.08
18.	Octan-1-ol ^S^	1076	-	0.18 ± 0.01
19.	Nonan-2-one ^S^	1094	-	0.95 ± 0.03
20.	(*E,Z*)-Octa-3,5-dien-2-one	1095	-	0.20 ± 0.01
21.	Nonanal ^S^	1106	0.52 ± 0.02	3.48 ± 0.08
22.	2,6-Dimethylcyclohexanol ^S^	1111	-	0.74 ± 0.02
23.	4-Ketoisophorone ^S^	1148	0.89 ± 0.03	1.36 ± 0.05
24.	(*E,E*)-Nona-2,6-dienal ^S^	1157	-	0.09 ± 0.01
25.	5-Methylundecane	1159	-	0.14 ± 0.01
26.	(*E*)-Non-2-enal ^S^	1163	-	0.26 ± 0.01
27.	Dodec-1-ene ^S^	1193	0.03 ± 0.01	0.20 ± 0.03
28.	Decanal ^S^	1207	-	0.27 ± 0.02
29.	β-Cyclocitral ^S^	1224	0.15 ± 0.01	0.75 ± 0.04
30.	Benzothiazole ^S^	1228	0.14 ± 0.01	0.27 ± 0.01
31.	Benzenepropanenitrile	1245	-	0.45 ± 0.03
32.	β-Homocyclocitral ^S^	1260	-	0.31 ± 0.02
33.	(*E*)-Dec-2-enal ^S^	1265	3.48 ± 0.05	1.34 ± 0.07
34.	(*E,Z*)-Deca-2,4-dienal ^S^	1295	0.32 ± 0.01	0.51 ± 0.02
35.	1H-Indole ^S^	1301	0.15 ± 0.01	0.01 ± 0.01
36.	Undecanal ^S^	1308	-	0.64 ± 0.03
37.	(*E,E*)-Deca-2,4-dienal ^S^	1320	0.66 ± 0.01	2.23 ± 0.04
38.	Tetradec-1-ene ^S^	1393	0.12 ± 0.01	-
39.	Dodecanal ^S^	1410	-	3.16 ± 0.01
40.	α-Ionone ^S^	1430	-	0.79 ± 0.01
41.	(*E*)-Geranylacetone ^S^	1455	-	0.50 ± 0.02
42.	Dodecan-1-ol ^S^	1480	-	1.47 ± 0.05
43.	β-Ionone ^S^		0.77 ± 0.04	5.80 ± 0.06
44.	Pentadec-1-ene ^S^	1493	19.32 ± 1.21	1.54 ± 0.02
45.	Pentadecane ^S^	1500	5.87 ± 0.04	3.28 ± 0.03
46.	Tridecanal ^S^	1511	2.67 ± 0.01	18.51 ± 1.10
47.	(*E*)-Pentadec-7-ene	1517	8.35 ± 0.12	3.16 ± 0.08
48.	Tridecan-1-ol ^S^	1581	-	0.59 ± 0.02
49.	Tetradecanal ^S^	1614	0.51 ± 0.01	1.01 ± 0.05
50.	(*E*)-Heptadec-8-ene	1679	0.61 ± 0.01	-
51.	γ-Dodecalactone ^S^	1681	3.31 ± 0.08	2.20 ± 0.21
52.	(*Z*)-Heptadec-3-ene	1688	6.07 ± 0.51	1.23 ± 0.11
53.	Heptadec-1-ene ^S^	1694	5.05 ± 0.51	0.91 ± 0.02
54.	Heptadecane ^S^	1700	0.95 ± 0.01	-
55.	Pentadecanal ^S^	1716	1.34 ± 0.22	4.09 ± 0.11
56.	Tetradecanoic acid ^S^	1778	3.07 ± 0.03	4.77 ± 0.11
57.	Hexahydrofarnesyl acetone ^S^	1848	2.23 ± 0.11	1.59 ± 0.10
58.	Hexadecanoic acid ^S^	1973	-	2.69 ± 0.08
59.	Hexadecanal ^S^	1917	1.31 ± 0.04	-
60.	Dibutyl phthalate ^S^	1961	0.33 ± 0.05	0.20 ± 0.01
61.	(*Z,Z,Z*)-Octadeca-9,12,15-trien-1-ol (Linolenic alcohol)	2042	12.89 ± 1.01	5.08 ± 0.21
62.	Phytol ^S^	2115	7.19 ± 0.82	4.06 ± 0.11

E-the composition of volatile oil from the fresh sample; F-the composition of volatile oil composition from the air-dried sample; RI- retention indices determined relative to the alkanes C_9_–C_25_; Av-average area percentage determined by GC-FID/MS of 3 replicates; SD-the area percentages standard deviation for 3 replicates; ^S^-identification confirmed by standard compound; - the compound was not detected.

**Table 3:** The composition of fatty acids of *F. virsoides* investigated by GC-FID.

No.		Fatty Acid	Av ± SD (%)
1.	C12:0	Lauric acid ^S^	0.06 ± 0.01
2.	C14:0	Myristic acid ^S^	10.51 ± 0.07
3.	C15:0	Pentadecyclic acid ^S^	0.18 ± 0.00
4.	C16:0	Palmitic acid ^S^	8.10 ± 0.07
5.	C18:0	Stearic acid ^S^	1.35 ± 0.02
6.	C20:0	Arachidic acid ^S^	0.43 ± 0.11
7.	C24:0	Lignoceric acid ^S^	1.42 ± 0.33
		**Total saturated fatty acids (SFA)**	**22.03**
8.	C14:1	Myristoleic acid ^S^	0.20 ± 0.00
9.	C16:1	Palmitoleic acid ^S^	0.99 ± 0.07
10.	C18:1n9t + C18:1n9c	*cis*-Oleic acid+*trans*-Oleic acid ^S^	42.28 ± 0.24
11.	C20:1n9	Eicosenoic acid ^S^	0.65 ± 0.10
12.	C22:1n9	Erucic acid ^S^	0.32 ± 0.01
		**Total monounsaturated fatty acids (MUFA)**	**44.43**
13.	C18:2n6c	*cis*-Linoleic acid ^S^	6.51 ± 0.07
14.	C18:3n3	α-linolenic acid ^S^	2.13 ± 0.93
15.	C18:3n6	γ-Linolenic acid ^S^	0.42 ± 0.02
16.	C20:2n6	Eicosadienoic acid ^S^	1.51 ± 0.08
17.	C20:3n3	Eicosatrienoic acid ^S^	0.26 ± 0.06
18.	C20:3n6 (DGLA)	Dihomo-γ-linolenic acid ^S^	2.96 ± 0.03
19.	C20:4n6	Arachidonic acid ^S^	15.00 ± 0.05
20.	C20:5n3 (EPA)	Eicosapentaenoic acid ^S^	3.71 ± 0.02
21.	C22:2	Docosadienoic acid ^S^	1.24 ± 0.04
		**Total polyunsaturated fatty acids (PUFA)**	**33.71**
		**Total ω3 fatty acids**	**6.09**
		**Total ω6 fatty acids**	**27.62**

Av-an average percentage (%) of 3 replicates; SD-standard deviation; ^S^-identification confirmed by standard compound.

**Table 4:** Major non-volatile compounds in F3 and F4 and their tentative identification by HPLC-ESI(+)-HRMS.

No.	Compound	Rt (min)	Elemental Composition	*m*/*z* (Δ, ppm)	Peak Area (Arbitrary Units)	
					F3	F4
1.	1,3-Dihydroxy-2-propanyl 5,8,11,14-icosatetraenoate	13.70	C_23_H_38_O_4_	379.2844 (0.3)	2045	26
2.	2,3-Dihydroxypropyl palmitate	14.05	C_19_H_38_O_4_	331.2843 (0.0)	13,300	19,400
3.	2,3-Dihydroxypropyl 9-octadecenoate	14.34	C_21_H_40_O_4_	357.2998 (−0.4)	9240	330
4.	2,3-Dihydroxypropyl stearate	15.14	C_21_H_42_O_4_	359.3154 (−0.5)	27,100	17,900
5.	Isoamijiol oxidation product *	14.55	C_20_H_30_O_2_	303.2312 (−2.2)	1100	610
6.	(3a*R*,4a*R*,6*S*,8a*R*)-1-Isopropyl-3a,8a-dimethyl-5-methylene-2,3a,4,5,6,7,8,8a,9,10-decahydrobenzo[f]azulene-4a,6(3H)-diol (Isoamijiol)	14.54	C_20_H_32_O_2_	305.2469 (−2.0)	1800	60
7.	Fucoxanthin	15.50	C_42_H_58_O_6_	659.4299 (−1.1)	1340	10
8.	2-Hydroxypropyl palmitate	15.55	C_19_H_38_O_3_	315.2884 (−3.1)	197	788
9.	2-Hydroxypropyl stearate	16.55	C_21_H_42_O_3_	343.3200 (−2.0)	482	1690
10.	(3β,6α)-14-Methylergosta-8,24(28)-diene-3,6-diol (few isomers) **	16.25	C_29_H_48_O_2_	429.3723 (−0.9)	220	18
11.	13-Docosenamide	16.67	C_22_H_43_NO	338.3412 (−1.6)	7550	3650
12.	(2*E*)-3-[21-(Methoxycarbonyl)-4,8,13,18-tetramethyl-20-oxo-9,14-divinyl-3,4-didehydro-3-24,25-dihydrophorbinyl]acrylic acid	16.71	C_35_H_30_N_4_O_5_	587.2273 (−2.7)	1820	64
13.	Pheophorbide *a*	16.73	C_35_H_36_N_4_O_5_	593.2741 (−2.9)	1430	106
14.	3-[(21*R*)-21-(Methoxycarbonyl)-4,8,13,18-tetramethyl-20-oxo-9,14-divinyl-3,4-didehydro-3--24,25-dihydrophorbinyl]propanoic acid	16.77	C_35_H_32_N_4_O_5_	589.2422 (−4.0)	1240	62
15.	(3β)-3-Hydroxystigmast-5-en-7-one	18.22	C_29_H_48_O_2_	429.3720 (−1.6)	6380	205
16.	(3β,20*R*,22*E*,24*S*)-Stigmasta-5,22-dien-3-ol (β-Stigmasterol)	18.39	C_29_H_48_O	395.3664 *** (−2.1)	1950	28,670
17.	(2*S*)-1-Hydroxy-3-(tetradecanoyloxy)-2-propanyl (9*Z*)-9-octadecenoate	20.29	C_35_H_66_O_5_	567.4972 (−1.9)	849	2410
18.	3-Hydroxy-1,2-propanediyl bis(9-octadecenoate)	21.10	C_39_H_72_O_5_	621.5435 (−2.8)	66	1270
19.	3-Hydroxy-2-(palmitoyloxy)propyl stearate	21.60	C_37_H_72_O_5_	597.5433 (−3.3)	229	309
20.	Methyl (3*R*,10*Z*,14*Z*,20*Z*,22*S*,23*S*)-12-ethyl-3-hydroxy-13,18,22,27-tetramethyl-5-oxo-23-(3-oxo-3-{[(2*E*,7*R*,11*R*)-3,7,11,15-tetramethyl-2-hexadecen-1-yl]oxy}propyl)-17-vinyl-4-oxa-8,24,25,26-tetraazahexacycl; o[19.2.1.16,9.111,14.116,19.02,7]heptacosa-1(24),2(7),6(27),8,10,12,14,16,18,20-decaene-3-carboxylate	21.56	C_55_H_74_N_4_O_7_	903.5610 (−2.2)	-	1970
21.	3-Phorbinepropanoic acid, 9-acetyl-14-ethylidene-13,14-dihydro-21-(methoxycarbonyl)-4,8,13,18-tetramethyl-20-oxo-, 3,7,11,15-tetramethyl-2-hexadecen-1-yl ester	22.48	C_55_H_74_N_4_O_6_	887.5661 (−2.3)	17	2960
22.	3-Phorbinepropanoic acid, 3,4-didehydro-9-ethenyl-14-ethyl-24,25-dihydro-21-(methoxycarbonyl)-4,8,13,18-tetramethyl-20-oxo-, (2*E*)-3,7,11,15-tetramethyl-2-hexadecen-1-yl ester	22.53	C_55_H_72_N_4_O_5_	869.5550 (−2.9)	-	300
23.	Pheophytin *a*	22.75	C_55_H_74_N_4_O_5_	871.5711 (−2.4)	288	20,600

*-exact compound not determined; **-few lower intensity peaks of isomers were observed in the chromatogram; ***-dehydrated molecule [M-H_2_O+H]^+^; - the compound was not detected.

## Data Availability

Data are avail be for the authors for limited time.
